# Diagnostic Performance of EUS-Guided Sampling in Indeterminate Radiological Diagnosis of Pancreatic Disease and Intra-Abdominal Lymphadenopathy

**DOI:** 10.3390/jcm10173850

**Published:** 2021-08-27

**Authors:** Tae Yeon Jeon, Sung-Hoon Moon, Jong Hyeok Kim, Hyun Lim, Ho Suk Kang, Ji-Won Park, Sung-Eun Kim, Soo Kee Min

**Affiliations:** 1 Department of Radiology and Center for Imaging Science, Samsung Medical Center, Sungkyunkwan University School of Medicine, Seoul 06531, Korea; hathor97.jeon@samsung.com; 2 Department of Internal Medicine, Hallym University Sacred Heart Hospital, Hallym University College of Medicine, Anyang 14068, Korea; kjh825@hallym.or.kr (J.H.K.); hlim77@hallym.or.kr (H.L.); hskang76@hallym.or.kr (H.S.K.); miunorijw@hallym.or.kr (J.-W.P.); sekim@hallym.or.kr (S.-E.K.); 3 Department of Pathology, Hallym University Sacred Heart Hospital, Hallym University College of Medicine, Anyang 14068, Korea; tgmsk@hallym.or.kr

**Keywords:** pancreas, diagnosis, endoscopic ultrasound

## Abstract

Background: Endoscopic ultrasound (EUS)-guided sampling has been widely used for pathologic diagnosis of pancreatic lesions and intra-abdominal lymphadenopathy. However, its effectiveness for diagnostic decision making in indeterminate radiological diagnosis has not been well determined. Materials and Methods: From March 2012 to October 2015, 98 consecutive patients who underwent EUS-guided FNA for solid intra-abdominal lesions were retrospectively analyzed (100 procedures). The purpose of EUS-guided sampling was classified as (1) confirmation of a high-confidence radiological diagnosis (High-confidence group) or (2) decision making in the differential diagnostic dilemma for indeterminate radiological diagnosis (Indeterminate group). The accuracies of EUS-guided sampling according to the purpose were analyzed and then compared. Results: Of the 100 procedures, 22 procedures (22%) came under the Indeterminate group, whereas 78 came under the High-confidence group. The accuracies did not differ between the Indeterminate and the High-confidence groups (86.4% vs. 88.5%, *p* = 1.000). Clinical conditions that required EUS-guided sampling for indeterminate radiological diagnosis were (1) pancreatic cancer vs. benign disease (*n* = 8; e.g., pancreatic cancer vs. mass-forming pancreatitis), (2) recurrence of previous/pre-existing cancer vs. benign disease (*n* = 5; e.g., recurrent gastric cancer vs. reactive lymph node), (3) pathologic differentiation of presumed malignancy (*n* = 6; e.g., lymphadenopathies in the previous history of esophageal cancer and colon cancer), or (4) miscellaneous (*n* = 3; e.g., tuberculous lymphadenopathy vs. other condition). Conclusions: EUS-guided sampling demonstrated an accuracy of 86.4% in the clinical setting of indeterminate radiological diagnosis, which was not different from that of the confirmation of high-confidence diagnosis.

## 1. Introduction

Endoscopic ultrasound (EUS)-guided sampling has been established as an effective procedure for the pathological diagnosis of solid pancreatic masses and peri-gastrointestinal lymphadenopathies [1,2,3,4]. Pathological diagnoses are mainly performed in clinical practice for (1) confirming the specific radiological diagnosis with high confidence (e.g., tissue diagnosis of presumed unresectable pancreatic cancer for guiding chemotherapy) or (2) differentiation of malignant from benign disease (e.g., mass-forming autoimmune pancreatitis vs. pancreatic cancer) [1,5]. EUS-guided sampling may influence decision making in the latter more than in former cases and may eventually alter management plans for patients.

EUS-guided sampling has shown good diagnostic performance in patients with solid pancreatic neoplasms, with a sensitivity of 85% and specificity of 98% [1]. Previous studies that addressed the effectiveness of EUS-guided sampling dealt with the overall ability in terms of tumor type, such as pancreatic adenocarcinoma or neuroendocrine tumor [1,6,7,8], but these studies did not discriminate the purpose of EUS-guided sampling (i.e., confirming vs. differentiating). The value of EUS-guided sampling may rely on the ability to differentiate ambivalent imaging diagnosis in clinical practice.

The present study aimed to evaluate the diagnostic performance of EUS-guided sampling for pancreatic masses and intra-abdominal lymphadenopathies in the setting of indeterminate radiological diagnosis, compared with the confirmation of a high-confidence radiological diagnosis. We also wanted to describe the clinical setting of indeterminate radiological diagnosis that needs EUS-guided sampling of solid pancreatic lesions or intra-abdominal lymph nodes.

## 2. Materials and Methods

### 2.1. Patients

This retrospective study was conducted according to the guidelines of the Declaration of Helsinki and approved by the Institutional Review Board of Hallym University Sacred Heart Hospital (2016-I141). We reviewed the medical records of patients who underwent EUS-guided sampling (fine-needle aspiration (FNA) or biopsy) to evaluate solid pancreatic lesions or intra-abdominal lymphadenopathies from March 2012 to October 2015 at Hallym University Sacred Heart Hospital, Anyang, Korea. The review of medical records included the purpose of EUS-guided sampling, location of the lesion (i.e., pancreas or lymph node), size of the targeted lesion, type of needle used (conventional 19-gauge, 22-gauge, or 25-gauge FNA needle, or Procore needle), occurrence of complications, and final diagnosis. The final diagnosis was based on surgical pathology or clinical follow-up of at least 6 months.

### 2.2. Classification of the Purpose of EUS-Guided Sampling

The purpose of EUS-guided sampling was determined after a review of the clinical data and radiologic images by consensus of a board-certified gastroenterologist (S.H.M.) and radiologist (T.Y.J.). The cases were classified as High-confidence group or Indeterminate group based on the following criteria. The experienced radiologist reviewed the radiological images (computed tomography or magnetic resonance imaging) and rated the confidence scale as high-confidence (i.e., definite specific malignancy) or indeterminate. The experienced gastroenterologists reviewed medical records and confirmed the purpose of the EUS-guided sampling. Any disagreement on the purpose of EUS-guided sampling was resolved by consensus between two investigators. When EUS-guided sampling was performed in the case with a high-confidence radiological diagnosis of malignancy (e.g., tissue diagnosis of presumed unresectable pancreatic cancer for guiding chemotherapy), the case was designated to the High-confidence group. Indeterminate radiological diagnosis included (1) indeterminate diagnosis of benign disease and (2) the need for differentiation of the type of malignancy. When EUS-guided sampling was performed in the case with an indeterminate radiological diagnosis for decision making (e.g., differentiating pancreatic cancer from mass-forming autoimmune pancreatitis), the case was designated to the Indeterminate group.

### 2.3. EUS-Guided Sampling Technique

All EUS-guided sampling procedures were performed by one of two experienced endosonographers (S.H.M. and J.H.K.). Patients were sedated with midazolam or propofol titrated to patient comfort. EUS-guided sampling was performed using a linear echoendoscope (GF-UCT 240, Olympus, Tokyo, Japan). The optimal puncture site was determined, and then a puncture was performed under the guidance of real-time EUS using a 19-gauge (Echo-19, Cook Endoscopy, Winston-Salem, NC, USA), 22-gauge (Echo-3-22, Cook Endoscopy), 25-gauge aspiration (Echo-25, Cook Endoscopy), or Procore (Echotip Procore, Cook Endoscopy) needle. The choice of needle type was made by the endosonographers based on the location, size, and vasculature of the targeted lesion. A transduodenal puncture was usually performed for a pancreatic head lesion, whereas a transgastric puncture was used for a pancreatic body/tail lesion. The mass was punctured, the stylet was removed, and suction was applied with a 10 mL syringe while moving the FNA needle within the lesion. By contrast, capillary sampling with the stylet slow-pull technique was used when using the Procore needle. The back-and-forth movement for aspiration was made more than 15 times per needle pass, usually with a fanning technique. The sampling procedure was repeated until a sufficient amount of specimen was obtained, as determined by gross visual inspection by the endosonographers. Aspirated material was expelled onto glass slides by stylet reinsertion, and smears were then made by the endosonographers. The smear slides were then fixed with 99% ethanol for cytological analysis. When using the Procore needle, a visible core was placed into a formalin bottle for histological analysis.

### 2.4. Statistical Analysis

Statistical analyses were conducted using software SPSS, version 23.0 software (SPSS, Chicago, IL, USA). Data were considered significant at *p* < 0.05. The 95% confidence interval for diagnostic performance was estimated by using Wilson’s method [9]. The diagnostic performance between Indeterminate and High-confidence groups was compared using a randomization test. Statistical differences were compared using Fisher’s exact test for categorical variables and the Mann–Whitney test for continuous variables.

## 3. Results

### 3.1. Baseline Characteristics

EUS-guided sampling for solid pancreatic lesions (*n* = 90) or intra-abdominal lymphadenopathies (*n* = 12) was performed in 100 consecutive patients (102 procedures) during the study period. The median age of patients was 66 years (male = 51 and female = 48 cases). The median lesion size was 35 mm (range 9–130 mm). The sampling needle used was predominantly a conventional 22-gauge FNA needle (*n* = 60), whereas a conventional 25-gauge FNA (*n* = 19), Procore 22-gauge (*n* = 13), Procore 19-gauge (*n* = 7), or conventional 19-gauge FNA (*n* = 3) needle was occasionally used. No acute complications of clinical importance arose, such as bleeding or pancreatitis.

Among the 100 patients, 88 were finally diagnosed as having a malignant neoplasm such as pancreatic cancer (*n* = 80), lymphoma (*n* = 3), neuroendocrine carcinoma (*n* = 1), gallbladder cancer (*n* = 1), recurrence of gastric cancer (*n* = 1), recurrence of pancreatic cancer (*n* = 1), and recurrence of esophageal cancer (*n* = 1). The remaining ten patients were finally diagnosed as having a benign disease such as autoimmune pancreatitis (*n* = 5), solid serous cystadenoma of the pancreas (*n* = 1), reactive lymph node (*n* = 2), and tuberculous lymphadenopathy (*n* = 2). Two patients were lost to follow-up and excluded. The final analysis included a total of 100 procedures from 98 patients.

### 3.2. EUS-Guided Sampling-Based Decision Making

**Figure 1:**
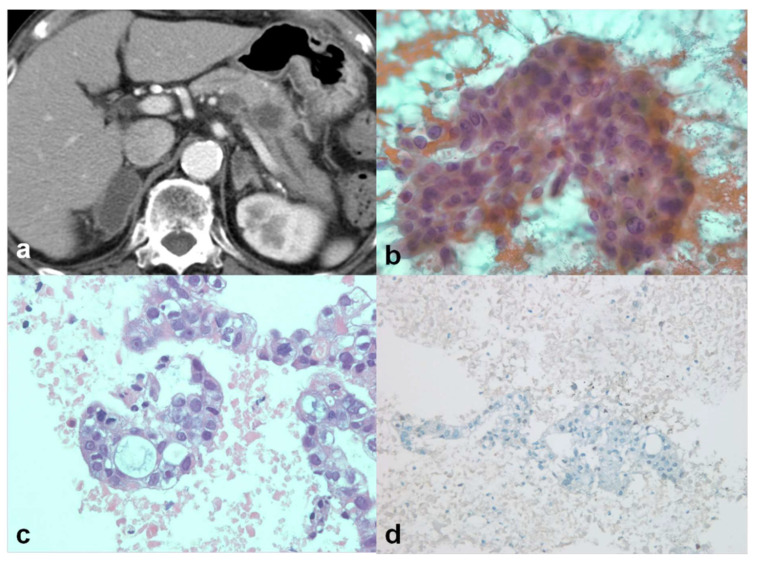
A 79-year-old male patient with previous history of lung cancer (patient no. 20). (**a**) CT scan showed pancreatic body mass with upstream duct dilatation. The differential diagnosis was primary pancreatic cancer versus metastasis from lung cancer. EUS-guided sampling was performed. (**b**) Cytologic examination demonstrated nuclear pleomorphism, prominent nucleoli, and mucin vacuole, those were consistent with adenocarcinoma (×400, Papanicolaou stain). (**c**) Histologic examination demonstrated similar findings (×400, hematoxylin and eosin stain). (**d**) Immunohistochemical stain with thyroid transcription factor 1 (TTF-1) showed a negative result, suggesting nonlung origin (×200).

**Figure 2:**
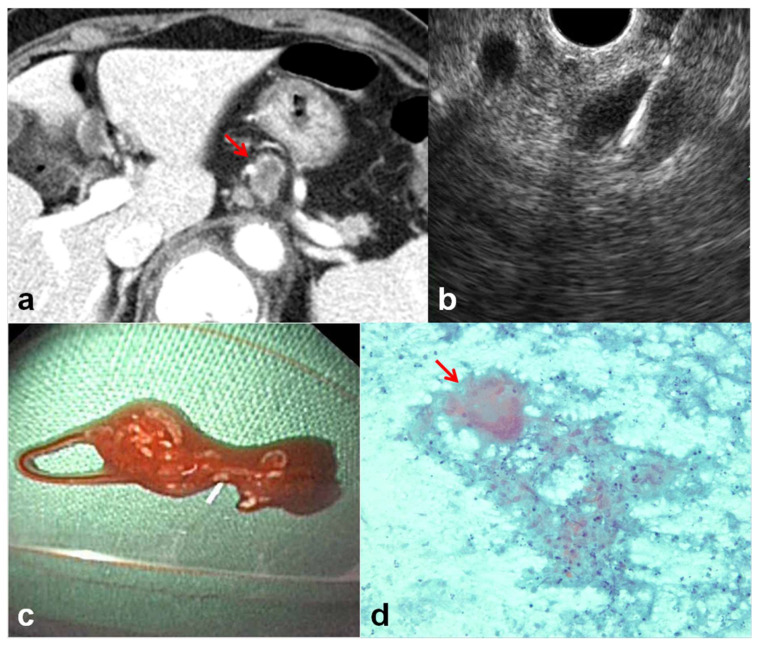
A 79-year-old female patient with multiple enlarged lymph nodes (patient no. 16). (**a**) CT scan showed multiple enlarged lymph nodes (arrow) in retroperitoneal spaces. The differential diagnosis was tuberculosis versus metastasis from other cancer. (**b**) EUS-guided sampling was performed. (**c**) Gross examination of aspirated material showed whitish granular material with blood. (**d**) Cytologic examination demonstrated necrotic background, multinucleated giant cell (arrow), and granuloma (×400, Papanicolaou stain). Polymerase chain reaction for tuberculosis was also positive.

Among 100 EUS-guided sampling procedures, 22 procedures (22%) came under the Indeterminate group, whereas 78 came under the High-confidence group by consensus of the gastroenterologist and radiologist, as described above. Clinical conditions that need EUS-guided sampling for indeterminate radiological diagnosis (decision making) were (1) pancreatic cancer vs. benign disease (*n* = 8; e.g., pancreatic cancer vs. mass-forming pancreatitis), (2) recurrence of previous/pre-existing cancer vs. benign disease (*n* = 5; e.g., recurrent gastric cancer vs. reactive lymph node), (3) pathologic differentiation of presumed malignancy (*n* = 6; e.g., lymphadenopathies in the previous history of esophageal cancer and colon cancer) (Figure 1), or (4) miscellaneous (*n* = 3; e.g., tuberculous lymphadenopathy vs. other condition) (Figure 2). The clinical data and final diagnoses are described in Table 1.

### 3.3. Diagnostic Performance between the Indeterminate Group and the High-Confidence Group

The median size of the lesion was 26 mm (interquartile range = 15–30 mm) in the Indeterminate group and 40 mm (interquartile range = 29–53 mm) in the High-confidence group (*p* < 0.0001). However, the accuracy was not significantly different between the Indeterminate and High-confidence groups (86.4% vs. 88.5%, *p* = 1.000). The sensitivity, specificity, positive predictive value, and negative predictive value for diagnosing malignancy were 75.0%, 100%, 100%, and 76.9% in the Indeterminate group and 88.4%, not available, 100%, and 0% in the High-confidence group (Table 2). The negative predictive value in the High-confidence group was 0% because this group did not have benign lesions. Lymph nodes were predominantly targeted by EUS for decision making (72.7%), whereas the pancreas was predominantly targeted for confirmation (85.2%) (*p* < 0.0001). The median size of the pancreas lesions was 39 mm (interquartile range = 27–47 mm), whereas the median size of the lymph node lesions was 22 mm (interquartile range = 12.5–26.5 mm) (*p* < 0.0001). The use of the Procore needle for acquiring core tissue did not significantly differ between the Indeterminate group and the High-confidence group (27.2% vs. 16.7%, *p* = 0.3547).

### 3.4. Effects of Type and Size of the Needle on Diagnostic Yield

The type of needle can be classified into conventional FNA needle and fine-needle biopsy (FNB) needle. When analyzing the diagnostic accuracies according to the type (FNA vs. FNB needle), diagnostic accuracies were 93.8% with the FNA needle and 61.5% with the FNB needle in the High-confidence group (*p* = 0.0052). In the Indeterminate group, the accuracies were 81.3% with the FNA needle and 100% with the FNB needle (*p* = 0.5325).

The diagnostic accuracies were also compared according to the size of the needle (19G vs. 22G needle). In the High-confidence group, the accuracies were 66.7% with the 19G needle and 91.1% with the 22G needle (*p* = 0.1323). In the Indeterminate group, the diagnostic accuracies were 100% with the 19G needle and 80.0% with the 22G needle (*p* = 1.0000).

## 4. Discussion

EUS-guided sampling has been widely used for the pathological confirmation of pancreatic tumors and metastatic lymph nodes [10]. This type of sampling for pancreatic masses has demonstrated a higher diagnostic accuracy when compared to CT-guided or ultrasound-guided FNA or ERCP-guided brush cytology [7]. A recent meta-analysis involving 31 articles documented that EUS-FNA has good accuracy for the diagnosis of pancreatic cancer, with a pooled sensitivity of 89% and specificity of 96% [11]. Nevertheless, despite the burgeoning literature on the accuracy of EUS-guided sampling in the diagnosis of pancreatic cancer, few studies have focused on the diagnostic accuracy of EUS-guided sampling in the clinical setting of the differential diagnostic dilemma.

Pancreatic ductal adenocarcinoma typically manifests at contrast-enhanced CT as a hypoattenuating mass or nodule, when compared with the pancreatic parenchyma; sometimes it is associated with interrupted pancreatic duct, dilated biliary and pancreatic ducts, atrophic upstream pancreatic parenchyma, and contour abnormality [12,13,14]. Although MRI could demonstrate an isoattenuating pancreatic adenocarcinoma on CT, a diagnostic uncertainty may persist after various imaging modalities [12]. Moreover, pancreatic diseases such as mass-forming chronic pancreatitis, lymphoma, and tuberculosis can mimic pancreatic ductal adenocarcinoma [15]. A pathologic examination based on EUS-guided sampling can resolve this type of diagnostic uncertainty. Our data showed that 22% of EUS-guided sampling procedures conducted on solid abdominal lesions were performed for decision making in the differential diagnostic dilemma (Indeterminate group), whereas 78% were performed only for confirmation of a particular radiological diagnosis (High-confidence group). The sensitivity of our EUS-guided sampling in the diagnosis of malignancy did not differ between the Indeterminate group and the High-confidence group (75.0% vs. 88.4%, *p* = 0.358).

Lymph nodes were predominantly targeted by EUS for decision making (72.7%) in our study. The European Society of Gastrointestinal Endoscopy clinical guideline states that EUS-FNA allows accurate determination of the nature of lymph nodes of unknown origin [10]. Yasuda et al. reported that the overall accuracy of EUS-FNA for unknown lymphadenopathy was 98% in 104 patients with mediastinal and intra-abdominal lymphadenopathy [16]. Our data showed lower accuracy (63.6%) than this previous report, possibly because our pathologist did not diagnose lymphoma when the small amount of lymphoid tissue was acquired by EUS-guided sampling.

The size of the lesion was significantly different between the Indeterminate group and the High-confidence group in our study (median = 26 mm vs. 40 mm, respectively; *p* < 0.0001). This difference in the lesion size between the two groups may reflect that the radiographic features present more typically in the advanced stage of the disease. Several studies have compared the diagnostic accuracy of EUS-guided sampling according to the lesion size. Haba et al. suggested that diagnostic accuracy was significantly lower for smaller lesions with thresholds of both 10 mm and 20 mm [8]. However, our data did not reveal any difference in diagnostic accuracy between the Indeterminate group and the High-confidence group (86.4% vs. 88.5%, *p* = 1.000). One plausible explanation is that the endoscopists might perform EUS-guided sampling more zealously in the Indeterminate group (e.g., increasing the number of strokes of the FNA needle).

The benign intra-abdominal diseases that needed EUS-guided sampling in our study included autoimmune pancreatitis and tuberculosis. EUS-guided fine-needle biopsy can provide a sample with the unique histopathological and immunohistochemical characteristics of autoimmune pancreatitis [3,5]. EUS-guided biopsy gave a pathological diagnosis of chronic pancreatitis with lymphoplasmacytic infiltration, fibrosis, and sclerosis in our patient with autoimmune pancreatitis. EUS-guided sampling is also helpful in distinguishing peripancreatic tuberculous lymphadenopathy masquerading as pancreatic malignancy [17]. In our patients with tuberculous lymphadenopathy, EUS-guided sampling gave findings of neutrophils, sometimes granulomas, positive tuberculous PCR, positive tuberculous culture, and no malignancy. As our study demonstrated the high accuracy in the Indeterminate group, the results of ‘negative for malignancy’ in the cases with the presumed benign disease can solidify the diagnosis of benign disease and may reassure clinicians during the follow-up periods.

Our study has several limitations. First, the purpose of EUS is retrospectively allocated into the Indeterminate group and the High-confidence group. The inherent bias caused by the retrospective design of our study should be highlighted when interpreting the study results. However, we tried to minimize the effect of the retrospective design by conducting consensus reviews by the gastroenterologist (medical recording) and radiologist (imaging study). A second limitation is the relatively small size of the study population. In addition, the indication and performance of EUS-guided sampling may vary from center to center because it depends on the expertise, local facilities, and clinical experience of physicians, endoscopists, and radiologists.

In conclusion, EUS-guided sampling demonstrated an accuracy of 86.4% in the clinical setting of diagnostic decision making for indeterminate radiological diagnosis. The accuracy did not differ between the Indeterminate and High-confidence groups. Lymph nodes were predominantly targeted by EUS in the indeterminate radiological diagnosis. The lesions that needed decision making based on EUS-guided sampling were smaller than those needing confirmation of a particular diagnosis. EUS-guided sampling may still be effective in solving the differential diagnostic dilemma.

## Figures and Tables

**Table 1:** Clinical data and final diagnosis of 21 patients who underwent EUS-guided sampling for decision making due to indeterminate radiological diagnosis.

Patient No.	Age	Sex	Clinical Setting	Needle Type	Size (mm)	Pathologic Result of EUS-Guided Sampling	Final Diagnosis
1	66	M	Pancreatic mass vs. serous cystadenoma	22G FNA	15	Inflammatory and lymphoid cells	Solid serous cystadenoma of the pancreas
2	57	M	Peripancreatic lymphadenopathy, S/P distal gastrectomy for advanced gastric cancer	25G FNA	13	Necrotic tissue with some macrophages	Reactive lymph node
3	70	F	Pancreatic duct stricture, mass-forming pancreatitis vs. pancreatic cancer	25G FNA	10	Atypia, favor reactive	Chronic pancreatitis
4	62	M	Lymphadenopathy near left gastric artery, S/P Whipple operation for pancreatic cancer	22G FNA	9	Suggestive of metastatic ductal adenocarcinoma	Recurrence of pancreatic cancer
5 *	66	F	Lymphadenopathy near common hepatic artery, S/P Distal gastrectomy for early gastric cancer	22G FNA	26	Fragments of bland-looking cells	Recurrence of gastric cancer
				22G FNA	26	Fragments of bland-looking cells	Recurrence of gastric cancer
6	45	M	Autoimmune pancreatitis vs. pancreatic cancer	19G FNB	35	Chronic pancreatitis with lymphocyte infiltration and fibrosis	Autoimmune pancreatitis
7	70	M	Pancreatic duct stricture, chronic pancreatitis vs. pancreatic cancer	22G FNA	15	Negative for malignancy	Chronic pancreatitis
8	75	F	Autoimmune pancreatitis vs. pancreatic cancer	22G FNA	30	Benign ductal epithelial cells	Pancreatic cancer
9	71	M	Atypical pancreatic cancer vs. lymphoma	19G FNB	90	Diffuse large B-cell lymphoma	Lymphoma
10	77	M	Chronic pancreatitis vs. pancreatic cancer	22G FNA	24	Negative for malignancy	Chronic pancreatitis
11	77	M	Peripancreatic lymphadenopathy, S/P bisegmentectomy for cholangiocarcinoma	22G FNA	10	Negative for malignancy	Reactive lymph node
12	77	M	Necrotic lymphadenopathy along hepatoduodenal ligament	19G FNB	42	Neutrophils and necrotic debris. PCR for tuberculosis (+)	Tuberculous lymphadenopathy
13	66	M	Pancreatic head mass, S/P Rectal cancer	22G FNA	28	Consistent with adenocarcinoma	Pancreatic cancer
14	62	F	Lymphadenopathy with gallbladder mass	25G FNA	27	Consistent with adenocarcinoma	Gallbladder cancer
15	66	M	Pancreatic mass, pancreatic cancer vs. inflammatory pseudotumor	22G FNA	18	Adenocarcinoma	Pancreatic cancer
16	79	F	Multiple enlarged lymph nodes in retroperitoneal space	22G FNA	22	Many inflammatory cells and granuloma in necrotic background, PCR for tuberculosis (+)	Tuberculous lymphadenopathy
17	60	M	Pancreatic cancer vs. autoimmune pancreatitis	22G FNA	37	Proliferation of activated lymphoid cells	Autoimmune pancreatitis
18	69	F	Pancreatic mass, H/O non-Hodgkin’s lymphoma	19G FNB	36	Adenocarcinoma	Pancreatic cancer
19	69	M	Periaortic lymphadenopathy, S/P Ivor-Lewis operation due to esophageal cancer, S/P colon cancer	22G FNA	19	Suggestive of squamous cell carcinoma	Recurrence of esophageal cancer
20	79	M	Pancreatic mass, H/O lung cancer	22G FNB	29	Adenocarcinoma favor pancreatic origin †	Pancreatic cancer
21	56	M	Pancreatic mass and lung mass	22G FNB	40	Favor neuroendocrine carcinoma €	Neuroendocrine carcinoma

PCR, polymerase chain reaction; FNA, fine-needle aspiration; FNB, fine-needle biopsy. * This patient underwent 2 EUS-guided FNA procedures. Both pathologic results showed benign nature cells and interferon-gamma release assay showed positive, so followed-up with antituberculosis medication. CT scan 3 months after EUS-guided sampling, the size of the lymph node increased, and percutaneous biopsy demonstrated adenocarcinoma. † Based on immunohistochemical results: TTF-1 (−), CK19(+), CK20 (+), CK7(+). € Immunohistochemical staining: Synaptophysin (+), CD56 (+), TTF-1: a few positive, P40 (−).

**Table 2:** Diagnostic performance to differentiate malignant and benign lesions between indeterminate radiological diagnosis group and high-confidence radiological diagnosis group.

	Indeterminate Group	High-Confidence Group	*p* Values
Sensitivity (95% CI)	75.0% (46.7–91.1%)	88.5% (79.5–93.8%)	0.358
Specificity (95% CI)	100% (72.3–100%)	N-A	N-A
Positive predictive value (95% CI)	100% (70.1–100%)	100% (94.7–100%)	1.000
Negative predictive value (95% CI)	76.9% (49.7–91.8%)	0% (0–30.0%)	<0.0001
Accuracy (95% CI)	86.4% (66.7–95.3%)	88.5% (79.5–93.8%)	1.000

## Data Availability

Not applicable.

## References

[1] Hewitt M.J., McPhail M.J., Possamai L., Dhar A., Vlavianos P., Monahan K.J. (2012). EUS-guided FNA for diagnosis of solid pancreatic neoplasms: A meta-analysis. Gastrointest. Endosc..

[2] Costache M.I., Iordache S., Karstensen J.G., Saftoiu A., Vilmann P. (2013). Endoscopic ultrasound-guided fine needle aspiration: From the past to the future. Endosc. Ultrasound.

[3] Yoon S.B., Moon S.H., Song T.J., Kim J.H., Kim M.H. (2020). Endoscopic ultrasound-guided fine needle aspiration versus biopsy for diagnosis of autoimmune pancreatitis: Systematic review and comparative meta-analysis. Dig. Endosc..

[4] Esposito I., Hruban R.H., Verbeke C., Terris B., Zamboni G., Scarpa A., Morohoshi T., Suda K., Luchini C., Klimstra D.S. (2020). Guidelines on the histopathology of chronic pancreatitis. Recommendations from the working group for the international consensus guidelines for chronic pancreatitis in collaboration with the International Association of Pancreatology, the American Pancreatic Association, the Japan Pancreas Society, and the European Pancreatic Club. Pancreatology.

[5] Moon S.H., Kim M.H. (2012). The role of endoscopy in the diagnosis of autoimmune pancreatitis. Gastrointest. Endosc..

[6] Chatzipantelis P., Salla C., Konstantinou P., Karoumpalis I., Sakellariou S., Doumani I. (2008). Endoscopic ultrasound-guided fine-needle aspiration cytology of pancreatic neuroendocrine tumors: A study of 48 cases. Cancer.

[7] Hartwig W., Schneider L., Diener M.K., Bergmann F., Buchler M.W., Werner J. (2009). Preoperative tissue diagnosis for tumours of the pancreas. Br. J. Surg..

[8] Haba S., Yamao K., Bhatia V., Mizuno N., Hara K., Hijioka S., Imaoka H., Niwa Y., Tajika M., Kondo S. (2013). Diagnostic ability and factors affecting accuracy of endoscopic ultrasound-guided fine needle aspiration for pancreatic solid lesions: Japanese large single center experience. J. Gastroenterol..

[9] Newcombe R.G. (1998). Two-sided confidence intervals for the single proportion: Comparison of seven methods. Stat. Med..

[10] Dumonceau J.M., Polkowski M., Larghi A., Vilmann P., Giovannini M., Frossard J.L., Heresbach D., Pujol B., Fernandez-Esparrach G., Vazquez-Sequeiros E. (2011). Indications, results, and clinical impact of endoscopic ultrasound (EUS)-guided sampling in gastroenterology: European Society of Gastrointestinal Endoscopy (ESGE) Clinical Guideline. Endoscopy.

[11] Chen G., Liu S., Zhao Y., Dai M., Zhang T. (2013). Diagnostic accuracy of endoscopic ultrasound-guided fine-needle aspiration for pancreatic cancer: A meta-analysis. Pancreatology.

[12] Kim J.H., Park S.H., Yu E.S., Kim M.H., Kim J., Byun J.H., Lee S.S., Hwang H.J., Hwang J.Y., Lee M.G. (2010). Visually isoattenuating pancreatic adenocarcinoma at dynamic-enhanced CT: Frequency, clinical and pathologic characteristics, and diagnosis at imaging examinations. Radiology.

[13] Prokesch R.W., Chow L.C., Beaulieu C.F., Bammer R., Jeffrey R.B. (2002). Isoattenuating pancreatic adenocarcinoma at multi-detector row CT: Secondary signs. Radiology.

[14] Sahani D.V., Shah Z.K., Catalano O.A., Boland G.W., Brugge W.R. (2008). Radiology of pancreatic adenocarcinoma: Current status of imaging. J. Gastroenterol. Hepatol..

[15] To’o K.J., Raman S.S., Yu N.C., Kim Y.J., Crawford T., Kadell B.M., Lu D.S. (2005). Pancreatic and peripancreatic diseases mimicking primary pancreatic neoplasia. Radiographics.

[16] Yasuda I., Tsurumi H., Omar S., Iwashita T., Kojima Y., Yamada T., Sawada M., Takami T., Moriwaki H., Soehendra N. (2006). Endoscopic ultrasound-guided fine-needle aspiration biopsy for lymphadenopathy of unknown origin. Endoscopy.

[17] Kim J.B., Lee S.S., Kim S.H., Byun J.H., Park D.H., Lee T.Y., Lee B.U., Jeong S.U., Seo D.W., Lee S.K. (2014). Peripancreatic tuberculous lymphadenopathy masquerading as pancreatic malignancy: A single-center experience. J. Gastroenterol. Hepatol..

